# Seafood Consumption Trends among U.S. Consumers: Influences of the COVID-19 Pandemic

**DOI:** 10.3390/foods13172682

**Published:** 2024-08-26

**Authors:** Lauren B. Errickson, Yanhong Jin, Douglas Zemeckis, William K. Hallman

**Affiliations:** 1 School of Environmental and Biological Sciences, Rutgers, The State University of New Jersey, 112 Log Cabin Road, North Brunswick, NJ 08902, USA; 2 Department of Agriculture, Food, and Resource Economics, Rutgers, The State University of New Jersey, 112 Log Cabin Road, North Brunswick, NJ 08902, USA; jinyh@sebs.rutgers.edu; 3 Department of Agriculture and Natural Resources, Rutgers Cooperative Extension, Rutgers, The State University of New Jersey, 112 Log Cabin Road, North Brunswick, NJ 08902, USA; zemeckis@njaes.rutgers.edu; 4 Department of Human Ecology, Rutgers, The State University of New Jersey, 112 Log Cabin Road, North Brunswick, NJ 08902, USA; hallman@sebs.rutgers.edu

**Keywords:** consumer preferences, food choice, seafood intake, Dietary Guidelines for Americans, COVID-19

## Abstract

The Dietary Guidelines for Americans (DGA) recommend twice-weekly consumption of seafood for health benefits, yet many U.S. consumers have historically fallen short of this target. The economic and societal impacts of the COVID-19 pandemic brought concern about further declines in seafood intake. This study evaluated the influence of COVID-19 on seafood intake among U.S. residents, toward understanding potential public health implications. A nationally representative cross-sectional survey of 1200 U.S. residents conducted in June 2021 evaluated the frequency and types of seafood consumed, and purchase methods used, before and during the early stages of the COVID-19 pandemic. The results (weighted data) indicate most U.S. consumers (90%) eat seafood, yet only 19% meet the seafood DGA. The likelihood of meeting the DGA was higher among consumers who were Asian, Black, or Hispanic; older; unmarried; of higher income; familiar with the DGA; choosing seafood for health reasons; and living in Atlantic or Gulf coast states. During COVID-19, some increased seafood intake for health reasons (45%), while others reduced intake due to factors such as cost (29%), limited availability (16%), and challenges with preparation (11%). Fresh salmon (68%), frozen shrimp (59%), and cooked oysters (41%) were most frequently purchased by consumers of these foods. More consumers indicated purchasing wild salmon (62%), shrimp (44%), and oysters (51%) than farm-raised products, though many were unsure. Fewer consumers included seafood in online grocery orders (36%) than meal kit orders (61%) when using these services. Though many consumers continued to eat seafood despite decreased restaurant patronage, most did not reach the intake level needed to maximize health benefits. Educational and marketing efforts promoting the health benefits of seafood and the convenience of procurement via online purchase methods may encourage intake across demographic groups to benefit public health outcomes in the U.S.

## 1. Introduction

Seafood represents an important dietary component for many U.S. residents, but the COVID-19 pandemic brought the potential for substantial shifts in seafood intake behavior. Seafood consumption has historically been influenced by complex factors that include health, economics, and convenience, among others. This study sought to understand the impact of the early stages of the pandemic on seafood intake frequency in the context of the Dietary Guidelines for Americans, as well as seafood purchasing patterns among U.S. consumers, as changes to either could have important implications for the seafood industry as well as for public health.

Seafood consumption is associated with numerous health benefits, including reduced risk and severity of cardiovascular disease [1], metabolic syndrome [2], and cognitive impairment with increasing age [3]. Consumption within appropriate levels has also been associated with improved neurological and cognitive development in youth [4]; a benefit attributed to the dietary intake of omega-3 fatty acids (specifically eicosapentaenoic acid and docosahexaenoic acid), as well as neuroprotective iodine and selenium [5]. For these reasons, the U.S. Dietary Guidelines for Americans (DGA) recommend that most adults eat at least eight ounces (227 g) of a variety of seafood per week [6] and the U.S. Food and Drug Administration (FDA) and U.S. Environmental Protection Agency (EPA) advise those who are or plan to become pregnant, or who are breastfeeding, to eat eight to twelve ounces (227–340 g) of varieties of seafood that are rich in omega-3 fatty acids but low in methylmercury [7]. Children are also recommended to consume age-appropriate amounts of seafood [7].

However, U.S. consumers have historically fallen short of meeting these recommendations [8,9]. According to the 2020–2025 DGA, almost 90% of the U.S. population aged one year old or above do not meet the dietary recommendations for seafood intake [6]. The Food Marketing Institute (FMI) reported that, in 2020, about 57% of American adults consumed fish at least once per month, but only 25% reported consuming fish twice per week in accordance with the DGA; 43% reported consuming little to no fish [10]. Thus, there exists a need for increased levels of seafood consumption to meet this dietary recommendation among the U.S. population.

Further, the majority of seafood eaten in the U.S. consists of relatively few types. In 2019, 74% of seafood (by weight) consumed in the U.S. comprised only 10 species, with shrimp, salmon, and canned tuna accounting for 52% of total seafood consumption [11]. In limiting their consumption to a small number of species, U.S. consumers may not be maximizing the nutritional benefits of seafood; simultaneously, they may be placing higher harvest pressure on a relatively small subset of seafood types.

The social and economic repercussions associated with the identification of the SARS-CoV-2 respiratory virus and the subsequent designation of the COVID-19 pandemic in March 2020 by the World Health Organization [12] had the potential to further limit the frequency and variety of seafood intake by U.S. consumers. Supply chain disruptions during the pandemic may have influenced consumers’ ability to purchase the seafood (and other items) they usually eat [13]. Reported declines in U.S. fresh seafood catches, imports, and exports [14] along with decreased restaurant patronage in early 2020 [14] indicated the emergence of a negative impact of the pandemic on the seafood sector. Before the COVID-19 pandemic, 65% of seafood expenditures occurred at restaurants [15]. The decline in restaurant patronage due to the temporary closure of restaurants, social distancing, and fear of infection during the pandemic could have significantly reduced already-low seafood intake levels by Americans. However, analysis of food sourcing data during the early stages of the pandemic by White et al. [14] indicates more consumers sought options for home seafood consumption than did so previously, and market data suggests a 28.4% increase in the value of grocery store purchases of fresh, frozen, and shelf-stable seafood items in 2020 [16]. One recent study of U.S. consumers suggests many shoppers did not change the type of seafood eaten pre- and post-pandemic, but they did consume more seafood at home compared to before the pandemic [17].

While the rise in at-home consumption may have offset the decline in restaurant-based seafood intake for some, the widespread economic impacts of the pandemic may have contributed to decreased seafood intake for others. Price has historically been recognized as a factor influencing the likelihood of seafood consumption [18]; financial hardships experienced during the pandemic could have contributed to the consumer selection of proteins that cost less than seafood options. Recent data suggest approximately 17% of European consumers decreased seafood intake during the 2020 lockdown periods [19], a trend that may also have occurred in the United States. Though market data and international consumer trends provide important insights about potential changes in seafood consumption among U.S. consumers, a direct assessment of consumer intake is warranted given the important implications for nutrition and public health outcomes, as well as the viability of the U.S. seafood industry.

Along with changes in eating habits, how consumers obtained groceries also shifted during the COVID-19 pandemic. Overall, U.S. consumers made less frequent trips to the grocery store [17,20], reduced in-store shopping time [20], and had the same [17] or higher [20] expenditures during each shopping trip. They reduced away-from-home (e.g., restaurant) food expenditures [21] and increased online grocery shopping during the early stages of the pandemic [20,21]. With online grocery orders projected to increase to over 21% of the market share by the year 2025 [22], the opportunity exists to increase seafood intake by consumers if they are amenable to purchasing seafood online for home consumption. However, the same study of U.S. consumers found they were less likely to include seafood in home delivery orders compared to other meals [17], suggesting that barriers to adoption exist.

To better understand current trends and potential changes in seafood consumption among U.S. consumers, this study had the following three objectives: (1) evaluate self-reported seafood intake behavior across demographic segments of the U.S. population, including changes that may have occurred during the COVID-19 pandemic; (2) assess seafood purchasing patterns, including the types of seafood bought and whether consumers utilized online methods to buy seafood, during the early stages of the pandemic; and (3) examine factors affecting the probability of meeting the seafood consumption recommendations set by the DGA.

## 2. Materials and Methods

To capture potential variability in consumer trends across different seafood types, this study evaluated self-reported intake and purchasing patterns regarding a finfish (salmon), a crustacean (shrimp), and a mollusk (oysters). Each seafood type was specifically selected for this study to represent a different sector of the seafood industry. In the U.S., salmon and shrimp are among the most consumed seafood types (by weight) and salmon, shrimp, and oysters are among the top five most valuable aquaculture species [23]. Additionally, both wild-caught and farm-raised salmon, shrimp, and oysters are available for purchase throughout the U.S., allowing for the inclusion of these two harvest methods. This study was approved on 10 September 2020 by the Institutional Review Board of Rutgers, the State University of New Jersey per study number PRO2020001880.

### 2.1. Survey Design

A survey was conducted in June 2021 with a nationally representative sample of U.S. adults to assess self-reported seafood intake and purchasing trends before (March 2019–March 2020) and during the early stages (March 2020–March 2021) of the COVID-19 pandemic. This cross-sectional survey was conducted online by the international research data and analytics company YouGov.com, which coordinated respondent enrollment from their existing panel of registered participants, soliciting them via email. Upon recruitment, YouGov.com administered the online survey, ensured survey completion, and provided raw anonymized data according to the specifications of the study investigators.

The survey instruments included dietary intake questions adapted from the National Health and Nutrition Examination Survey [24] to assess frequency of seafood consumption before and during the early stages of the COVID-19 pandemic, as well as to determine the type (e.g., salmon, shrimp, oysters, tuna), form (e.g., fresh, frozen, canned), and harvest method (i.e., wild-caught or farm-raised) of seafood eaten. Standardized questions routinely asked of the YouGov.com respondent panel were used to compile sociodemographic characteristics of the respondents. Additional survey questions were developed de novo to assess study-specific topics of interest, including reasons for modifying seafood intake and purchase behaviors during the COVID-19 pandemic. Key informant interviews were conducted with representatives of the U.S. seafood industry [25] prior to survey development to ensure relevance and content validity of de novo survey questions. Key informants representing seafood production, retail, research institutions, government agencies, industry associations, and a certifying agency across the salmon, shrimp, and oyster sectors participated in the interviews to represent broad content knowledge for validity of the de novo questions. The survey instrument in its entirety was pilot tested prior to data collection. The full questionnaire is available from the authors upon request.

### 2.2. Participants

The sample included 1200 U.S. consumers aged 18 years and above selected to participate from the YouGov.com U.S. survey panel, which comprises two million pre-screened respondents whose sociodemographic characteristics reflect that of U.S. residents. More information about the panel can be found at: https://today.yougov.com/about/panel, accessed on 11 August 2024. Participants were matched to sampling frames according to gender, race, age, and education level to yield a final weighted sample approximately representative of the U.S. adult population based on U.S. Census Data [26], permitting projections to the U.S. adult population with an approximate margin of error of +/−2.8% with a 95% level of confidence. Participants’ responses to initial questions about whether they, or anyone in their household, eat seafood served as a filter for whether they would be asked follow-up questions related to seafood intake and purchase. Specifically, participants from a household in which neither they nor any other household member eats seafood were excluded from more detailed survey questions regarding household seafood purchase preferences. Before completing the survey, participants provided their consent to participate in alignment with the protection of human subjects requirements of the institutional review board that reviewed this study. Confidentiality of participants was maintained by YouGov.com, which provided only deidentified data to the study investigators.

### 2.3. Statistical Analysis

Using weighted data to allow for the projection of results from survey respondents to the U.S. population, descriptive statistics were used to explore seafood consumption among U.S. consumers. Differences in adherence to the DGA before and during the pandemic were evaluated using McNemar’s Test, using a significance level of *p* < 0.05. Logistic regression was conducted to estimate the association between the contributing independent factors and the adherence to the DGA recommendation for seafood intake. Guided by our literature review, we included characteristics of the respondent (gender, marital status, race, age cohort, education level, employment status, religious affiliation, perceived benefits of seafood consumption, and familiarity with the DGA), as well as household factors (household income, having children under 5 or 5–18 years old, and coastal location based on state of residency: Atlantic, Pacific, Gulf, and Great Lakes) in the model. We also included province fixed effects, controlling for other potential variations at the state level that could affect DGA adherence. The variance–covariance matrix for the error term in the logistic model is clustered by coastal residency, accounting for correlation among respondents living in states with waterfront or coastal shorelines, but not among those without coastal residency.

Data analyses were conducted using IBM SPSS Statistics for Windows (version 27; IBM Corp., Armonk, NY, USA) and, for regression analyses, Stata Statistical Software (version 17; StataCorp LLC, College Station, TX, USA).

## 3. Results

### 3.1. Adherence to Seafood Intake Recommendations

The results show that, in most cases (96.3%), the survey respondents or a member of their household eat seafood (Table 1). Similarly, most individual U.S. adults (90.4%) personally eat seafood. However, many do not eat seafood with sufficient frequency to meet the recommendations of the Dietary Guidelines for Americans. During the early stages of the pandemic, 18.9% of U.S. adults met the DGA recommendation to eat seafood at least twice per week, a significant increase from pre-pandemic levels (16.6%) (McNemar’s asymptotic significance, *p* = 0.028). The logistic regression model explained nearly 14% of the variation in adherence to the DGA recommendation for seafood consumption among the general population of U.S. adults (Pseudo-R^2^ = 0.138; Table 2), with a corrective prediction ratio of 77.40%.

Since the estimated coefficients of the logit model did not completely reflect the marginal effect of the explanatory variables, we summarize both the estimated coefficients and the marginal effects along with the corresponding statistical significance in Table 2. A larger value and higher statistical significance for the marginal effect of an independent variable suggest a stronger and more significant association between the independent variable and adherence to the DGA. We found that higher income was associated with an increased likelihood of meeting the DGA recommendation for seafood consumption (*p* < 0.001) such that a $10,000 increase in income is associated with a half percentage point increase in the probability of DGA adherence. Respondents who were married were less likely to meet the DGA (*p* < 0.001) than those who were not married by five percentage points. Race is also an important predictor. Compared with non-Hispanic White participants, individuals who are Asian (*p* < 0.001), Black (*p* = 0.003), or Hispanic (*p* = 0.045) were more likely to meet the DGA by 14.1, 13.9, and 8.7 percentage points, respectively. Non-linear generational cohort differences were also observed regarding adherence to the DGA. Compared with the Silent Generation (age 76 years and above) and Gen Z (age 18–24 years), Millennials (age 25–40 years) were least likely to meet the DGA (*p* < 0.001, 9.1 percentage points less), followed by Baby Boomers (age 57–75 years; *p* < 0.001, 7.7 percentage points less), and Gen X (age 41–56 years; *p* = 0.064, 3.4 percentage points less). Those with less than a four-year college degree were more likely to meet the DGA (*p* < 0.001) by 10.1 percentage points compared with those with lower or higher education levels. Compared with residents of states with no coastal frontage or shoreline, residents of states bordering the Atlantic Ocean (*p* < 0.001) or the Great Lakes (*p* < 0.001) were more likely to meet the DGA by 10.9 and 6.0 percentage points, respectively; residents in states bordering the Pacific Ocean (*p* = 0.035) or Gulf of Mexico (*p* = 0.001) were less likely to meet the DGA by 1.4 and 6.4 percentage points, respectively.

Familiarity with the DGA also influences adherence to the seafood recommendations (Table 2). Compared with those who were not familiar with the DGA at all, respondents who self-reported being somewhat (*p* = 0.000) or very (*p* = 0.037) familiar with the DGA were significantly more likely to meet the seafood DGA by 2.5 and 9.4 percentage points, respectively. Eating seafood for health reasons and knowledge of how seafood may improve health also matters. Respondents who reported eating seafood for reasons of improved health (*p* = 0.000), agreed that seafood improves heart health (*p* = 0.003), or agreed specifically that seafood improves blood pressure (*p* = 0.066) were more likely to meet the DGA than those who did not. Marginal effects indicate the probability of the DGA adherence was higher by 2.0, 5.4, and 5.6 percentage points, respectively.

Adherence to the DGA for seafood was not significantly predicted by gender, religious affiliation, or by whether having children under the age of five, or between the ages of five and 18 years, in the household.

### 3.2. Changes in Seafood Intake during the COVID-19 Pandemic

A majority of respondents (73.1%) reported consuming seafood about as often during the early stages of the pandemic as they did pre-pandemic. However, 27% did indicate a change in seafood intake during the pandemic, with 13.9% reporting that they had consumed less and 13.0% consuming more than they did prior to the pandemic. The reasons cited most often for consuming less seafood included less frequent restaurant patronage and/or eating at home more frequently, followed closely by consumers reporting that they found seafood to be more expensive, and that they had less money available. Challenges preparing or obtaining seafood (due to lack of availability) were also noted as barriers to seafood intake during the pandemic (Table 3). Among those who consumed more seafood during the pandemic than before, 44.5% reported that they were doing so for the health benefits associated with increased seafood consumption. Some respondents also indicated they increased seafood consumption because they experienced financial improvement during the pandemic; others indicated increasing consumption because they were eating at home more often, eating more protein, adding variety to their diets, or trying new recipes (Table 3).

### 3.3. Home Seafood Consumption and Online Seafood Shopping

Among the respondents living in households in which they or someone else eats seafood (*n* = 1155), most (83.7%) reported purchasing seafood to prepare at home during the pandemic. The most frequently identified reason for not purchasing seafood for home preparation in these households was dislike of the required preparation (26.4%), followed by personal dislike of the taste (19.9%), the smell (15.7%) of seafood, or a family member’s dislike of its taste (9.0%) or smell (4.6%) (Table 4). Approximately 17.4% of these respondents also expressed concerns about the affordability of their preferred types of seafood. Some respondents also indicated challenges with seafood acquisition related to lack of availability (4.7%) and limited knowledge about how to select seafood (5.0%) (Table 4).

During the early stages of the pandemic, slightly more than half of the respondents from households in which someone eats seafood (50.6%) reported using at least one type of food or grocery pre-order or delivery service. Of the subset of participants who both live in households in which someone ate seafood and who ordered groceries for delivery and/or pickup (*n* = 412, 35.5%), almost two-thirds reported that their grocery order did contain seafood (64.0%). Of those in this subset who did not include seafood in their grocery order, a majority (66.6%) indicated not doing so because they prefer to select their seafood themselves, while others believed that the delivered seafood might be spoiled or damaged (29.5%), had other concerns (23.5%), or did not personally eat seafood (10.5%) (Table 5).

Only 5.5% of households in which someone eats seafood had ordered meal kits for delivery to their home during the early stages of the pandemic. However, 61.1% of these households did include seafood in their meal kit order(s). About one quarter (23%) of those who did not include seafood in their meal kit orders expressed concerns about spoiled or damaged seafood. Dislike of the seafood choices offered (29.1%) and lack of seafood availability in the meal kits (24%) also served as barriers to ordering meal kits that included seafood.

### 3.4. Types of Seafood Eaten during the Pandemic

**Figure 1:**
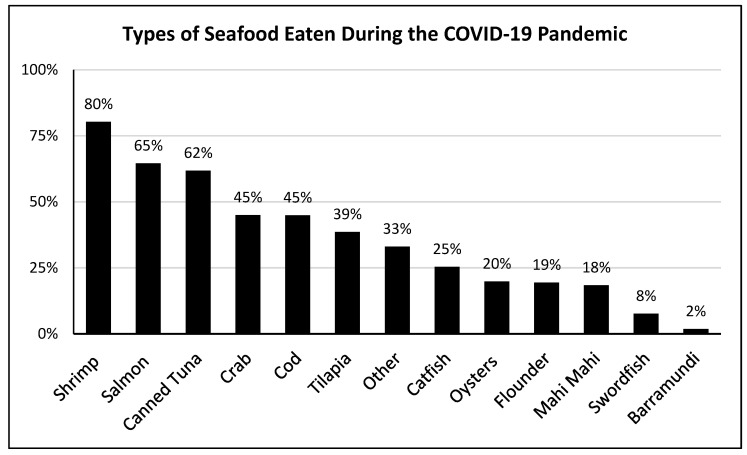
Percentage of seafood consumers eating various selections during the pandemic (weighted data; *n* = 1077).

**Figure 2:**
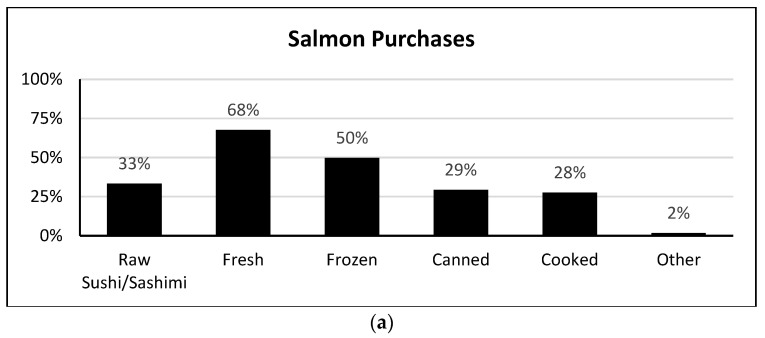

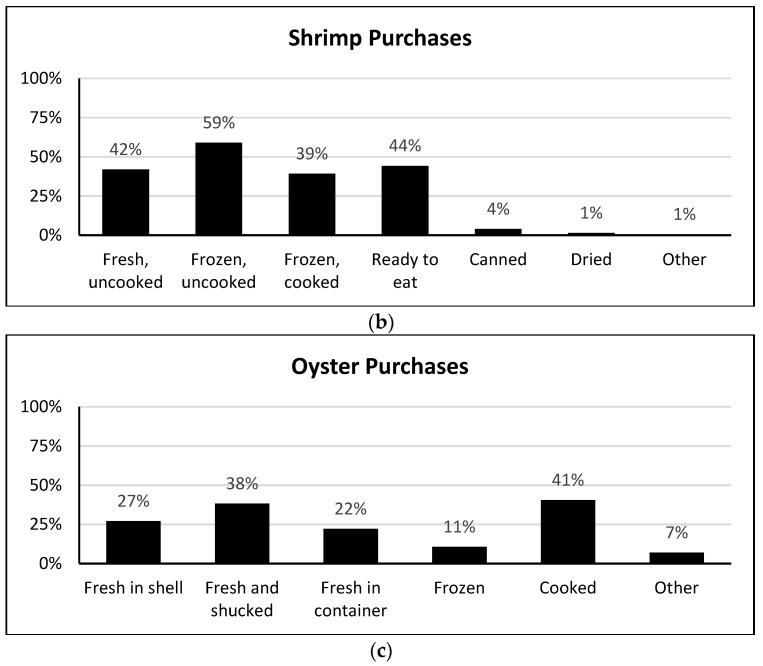
Forms of seafood purchased during the early stages of the COVID-19 pandemic (March 2020–March 2021) by U.S. residents who eat salmon (**a**), *n* = 694; shrimp (**b**), *n* = 865; and oysters (**c**), *n* = 213 (weighted data).

About eight in ten consumers reported eating shrimp, more than six in ten reported eating salmon, and more than half had eaten canned tuna during the pandemic (Figure 1). About one in five consumers reported having eaten oysters during the pandemic (Figure 1). When asked specifically about the forms of salmon, shrimp, and oysters they had purchased, the responses varied by type of seafood (Figure 2). The majority of consumers who purchased salmon purchased it fresh (67.6%) or frozen (49.7%) intending to cook it. One-third reported purchasing raw salmon as sushi/sashimi (33.3%). Those who purchased shrimp most often bought it uncooked and frozen (59.0%) or fresh (42.0%), though 44.3% also reported purchasing cooked, ready-to-eat shrimp. Oysters were most often purchased already cooked (40.5%) or fresh, shucked, and ready-to-eat (38.3%).

**Figure 3:**
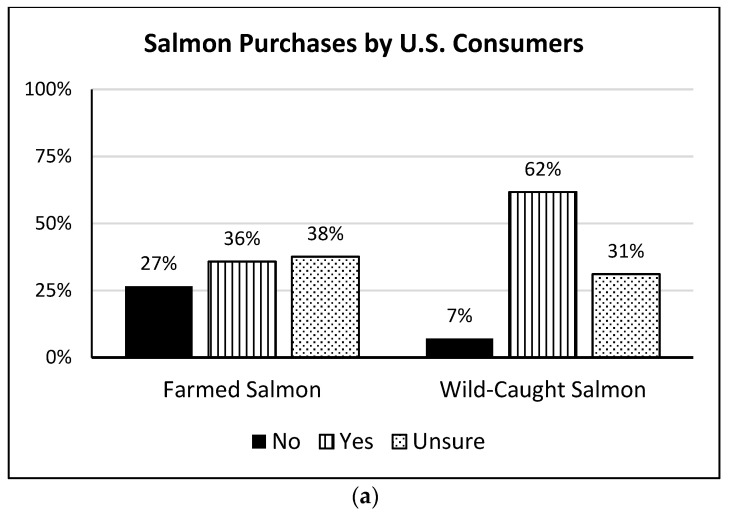

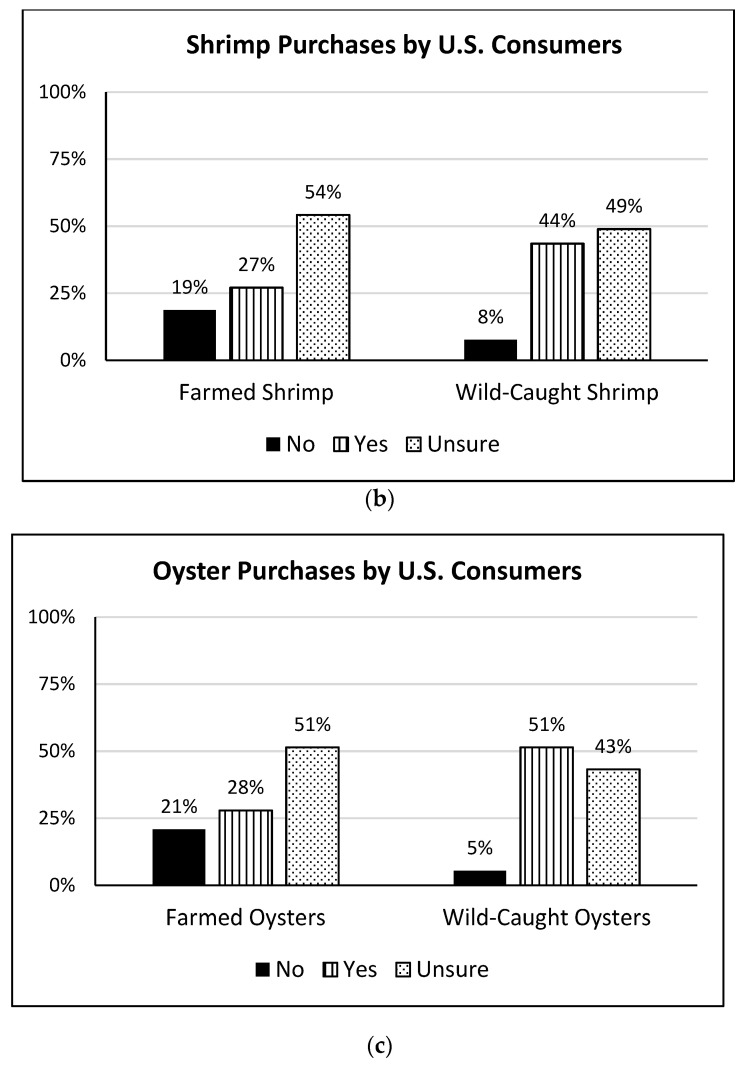
Reported purchases of farm-raised and/or wild-caught salmon (**a**), shrimp (**b**), and oysters (**c**) made during the early stages of the COVID-19 pandemic (March 2020–March 2021) by U.S. consumers who eat salmon (*n* = 694), shrimp (*n* = 865), and/or oysters (*n* = 213) (weighted data).

Among U.S. consumers who purchased salmon, shrimp, and oysters, more consumers reported having purchased wild-caught salmon (61.7%), shrimp (43.5%), and oysters (51.4%) than reported having purchased farm-raised products. However, across the three types of seafood, at least 30% of U.S. consumers indicated that they were unsure whether the seafood they had purchased was farm-raised or wild-caught (Figure 3).

## 4. Discussion

Despite concerns that the COVID-19 pandemic may have contributed to a decline in seafood intake among U.S. consumers, our results indicate that pre-pandemic consumption levels were generally maintained by most (73.1%) adults during the early stages of COVID-19. We found that some consumers ate seafood less frequently during the pandemic and that reasons included a decrease in restaurant patronage, aligning with reports by White et al. [14] that restaurant seafood demand decreased by about 70% during COVID-19 induced lockdown periods. We observed that consumers also reduced seafood intake frequency during the early stages of the pandemic due to expense, aligning with results documented by Engle et al. [17]. Even pre-pandemic, cost has been demonstrated to limit seafood intake [18,27]; that cost contributed to a decrease in seafood intake for some consumers during the early stages of the pandemic; therefore, it is not unexpected. Some consumers, however, ate seafood more frequently and even demonstrated increased adherence to the seafood DGA during the early stages of the pandemic. Nearly half (44.5%) of consumers who ate more seafood indicated they did so for health reasons; others justified doing so as a way to increase their dietary protein. These and similar reported results in the literature [17] indicate there is a potential to successfully encourage seafood intake from a health perspective in the U.S.

Demographic variability in seafood intake represents a complex interplay of multiple, intertwining factors. While it was beyond the scope of this study to confirm why specific demographic characteristics might explain intake behavior, our results contribute to a better understanding of who might be more or less likely to adhere to dietary guidance in the U.S. We found those more likely to meet the DGA reported higher levels of income and older age, as well as attainment of a four-year college degree. This aligns with the results of a recent review in which international seafood consumers were reportedly older and more affluent than non-consumers [27]. While Engle et al. recently found differences in consumption by age, education, and income, but not ethnic group [17] as others had demonstrated pre-pandemic [28], our results indicated consumers were also more likely to meet the DGA if they were Asian, Black, or Hispanic; or if they lived in a coastal or shoreline state bordering the Atlantic Ocean or the Great Lakes. These characteristics can serve as a basis for future, in-depth studies regarding the links between race, ethnicity, and seafood intake, and can perhaps guide interventional efforts toward consumers who are less likely to meet the DGA, which include younger, White, married members of the general population. Though our study only evaluated intake among U.S. adults, the 2020–2025 DGA indicate that nearly 90% of U.S. residents aged one year and above do not meet the seafood intake recommendations [6]. Future studies might consider expanding upon this work to evaluate seafood intake among children (those under age 18) to better understand demographic patterns in this sector of the U.S. population, which could serve as the foundation for developing household-level interventions to increase intake.

The results of this study suggest those among the general population who indicated familiarity with the DGA recommendations for seafood intake were more likely to meet them, as were those who agreed that cardiovascular benefits could result from eating seafood. In addition, specific reasons for greater levels of seafood intake cited by survey respondents suggest that at least some of the increases in home seafood consumption were influenced by growing concerns for overall personal health during the pandemic. Research supports seafood as a lean protein choice that provides important nutrients such as heart-healthy omega-3 fatty acids and zinc [6], which is linked to immune health [29]. Because of these demonstrated links between seafood intake and health, future studies should investigate the associations between consumer health status and adherence to the DGA. Additionally, as our results demonstrated that some consumers increased seafood intake for health reasons, future studies might investigate whether seafood education interventions that communicate the health benefits of seafood increase DGA adherence during later stages of, or after recovery from, the COVID-19 pandemic, especially if these interventions are directed toward demographic groups who currently tend not to meet the DGA.

The results of this study also show that some consumers navigated the early stages of the COVID-19 pandemic by cooking more often at home and may have capitalized on the opportunity to increase healthy dietary choices, including eating more seafood. Respondents also indicated a desire to expand the variety of foods in their diets. Both are trends that may continue as social reintegration and visits to restaurants and bars potentially return to pre-pandemic levels. It is possible that those who tried seafood at home during the pandemic might re-enforce their preference for seafood consumption and continue to expand their seafood selections while dining out even after the pandemic. Many households did purchase seafood to prepare at home during the pandemic; therefore, decreased restaurant patronage during the pandemic may have had a lesser impact on seafood intake than expected. Indeed, emerging research has confirmed increased retail sales as a counterbalance to the reduced levels of restaurant patronage, demonstrating the resilience of the U.S. seafood industry and supply chains through the pandemic [30].

Despite the percentage of consumers who reported increased seafood intake during the early stages of the pandemic, most consumers still do not meet the DGA recommendations for seafood. Nearly three-quarters of U.S. consumers did not change their seafood intake during the early stages of the pandemic. Pre-pandemic barriers such as dislike for the taste and smell of seafood persisted and were cited by respondents as deterrents to purchasing seafood to prepare at home during the pandemic. To improve upon the overall seafood DGA adherence among U.S. adults, a variety of creative strategies are likely needed. Meal kits could reduce certain barriers to seafood intake, specifically those related to the selection and preparation challenges for some consumers. Meal kits typically operate through subscription services, offering recipes and the pre-measured ingredients required to make them, delivered directly to households and thereby facilitating the cooking of meals at home. Though recent studies suggest an increase in meal kit popularity in the U.S. [31], our results suggest only a small percentage (5.5%) of households ordered a meal kit during the early stages of the pandemic. It is encouraging, though, that many (61.1%) of the households receiving meal kits did include seafood in our study. Because the pieces of seafood included in the meal kit are pre-selected for consumers, knowledge of how to choose seafood selections is not required of the consumer. Further, meal kits include preparation instructions that reduce the level of prior consumer knowledge required to prepare seafood selections, which may be especially important toward motivating consumers to try new types of seafood.

Grocery orders were even more prevalent than meal kit purchases during the early stages of the pandemic, with many consumers also including seafood in grocery orders. However, there remains a subset of consumers who did not include seafood in their grocery or meal kit orders, and these represent an opportunity for increased seafood sales and consumption. Addressing consumer concerns about the potential spoilage or damage of seafood identified in this study and reflected in other recent work [17] may expand consumer confidence in online seafood ordering for those who already buy groceries and/or meal kits online. Additionally, this may persuade consumers who have not yet entered the online grocery marketplace to try this approach. A further means to encourage participation in online seafood ordering could be to address the limited knowledge and preparation skills noted by consumers in this study and others [27] through the provision of easy-to-prepare seafood recipes with online orders, which many meal kit services already offer.

Increasing the volume of seafood distributed through grocery and meal kit orders has important implications for both the seafood industry, which would benefit from increased sales, and for public health as U.S. consumers receive higher levels of the beneficial nutrients found in seafood with increased consumption. As Stoll et al. [32] suggest, alternative seafood networks that provide direct-to-consumer options for seafood purchasing outside of major distribution chains may fill a void experienced when grocery shopping is reduced. Even as the COVID-19 pandemic recedes, online shopping trends observed with regard to grocery and meal kit orders may persist, as can the opportunity for increased seafood distribution through these channels.

White et al. [14] found online searches for seafood delivery, takeout, and recipes increased by an average of 270% during 2020 as compared to pre-pandemic levels, further supporting our suggestion that there is an opportunity for increasing seafood intake via online orders. Capitalizing on the growing grocery and meal kit home delivery sector may provide further opportunities to increase seafood intake. Meal kits and online grocery ordering was trending upward even before the COVID-19 pandemic, and many more consumers used these systems during the pandemic. Consistent with our estimation of the percentage of consumers using online grocery and/or meal kit delivery options during the pandemic, Wang et al. [20] reported that about half of U.S. consumers surveyed did not use curbside or doorstep grocery delivery during the pandemic. However, in their study (which was not limited to seafood), the primary reason for not using grocery delivery cited among consumers surveyed was lack of service availability [20].

The most frequently purchased forms of seafood differed across the three seafood types we evaluated: fresh salmon, frozen shrimp, and cooked oysters were preferred. This may indicate differences in how well various seafood types can perform in different retail markets. For instance, both cooked and uncooked frozen shrimp were popular among consumers who had eaten shrimp during the pandemic, but it is not known whether these frozen items would be desirable if included as meal kit components. A relatively similar percentage reported purchasing fresh shucked oysters (38%) as purchased cooked oysters (41%). Both options (shucked and cooked) imply preparation by someone other than the consumer who purchased them, indicating consumers may not wish to prepare this particular seafood product at home as often as other types. However, future studies should further evaluate consumer preferences with respect to consuming oysters at home given their unique characteristics, including the need to shuck oysters purchased whole (in-shell), to learn what barriers may exist in this regard.

Capitalizing on the observed trend of some consumers increasing seafood due to health reasons during the early stages of the COVID-19 pandemic, communicating to consumers who are health conscious and may be interested in adding variety to their diet that online seafood ordering presents an easy way to meet those goals could encourage intake. Further, consumers who do not know how to prepare seafood at home might be more willing to learn by engaging with a meal kit service. Based on the reported barriers to seafood ordering observed in this study and by Engle et al. [17], however, food safety and quality would be key to the success of this endeavor. Educational efforts designed to increase home seafood preparation skills may also be of use, and examples are already employed throughout the U.S., particularly in coastal states [33,34]. Expanded development of targeted efforts that teach consumers how to prepare the types of seafood that are most available and affordable to them may be particularly useful. For example, educational efforts might focus on teaching the preparation of frozen seafood which, based on the results of this study, already appeals to some consumers, likely contains an equivalent nutritional benefit to fresh selections [35], and may even be easier to select and prepare. Adding to these educational efforts, clear messages about the nutrition and health benefits of seafood intake might encourage consumption even further.

Finally, while many consumers select commonly available types of seafood to purchase (e.g., salmon, shrimp, and tuna), the diversity of seafood available in the U.S. offers ample opportunity for delicious, affordable choices. The results of this study demonstrate an interest among consumers in increased dietary variety. Capitalizing on this interest to expand the kinds of seafood featured in consumer education programs and marketing campaigns might increase seafood intake overall. It could also contribute to the environmental sustainability of aquatic systems by increasing the consumption of lesser-known types of seafood that are sustainable choices. While it was beyond the scope of this study to evaluate the effectiveness of interventions designed to increase seafood intake, our results might inform the direction of future studies in this regard.

Interestingly, many consumers were unsure as to whether the salmon, shrimp, and/or oysters they purchased were farmed or wild-caught products. It is possible that these consumers did not have a preference (or a choice) regarding the harvest or production method of the seafood available to them, but it could be the case that consumers were unable to easily determine the production method for their choices. That many consumers indicated having eaten wild-caught versus farmed salmon (62% vs. 36%), shrimp (44% vs. 27%), and oysters (51% vs. 28%) illustrates that a relatively high percentage of consumers believe they are eating wild-caught seafood. However, as much as 70% of salmon [36], 90% of shrimp [37], and 90% of oysters [38] eaten in the U.S. are farm raised. The discrepancy may indicate that an even larger percentage of consumers do not know whether their seafood is farm raised or wild caught than indicated via their survey responses, a trend observed and reported by Bacher [39]. Along with evaluating the consumer preference for wild-caught and farm-raised seafood, future studies might investigate whether certain wild-caught and farm-raised seafood labeling strategies are more impactful toward encouraging the purchase and intake of seafood by U.S. consumers.

The consumption of aquaculture products (farmed fish) can provide a healthy, sustainable way for U.S. consumers to meet dietary guidelines for seafood intake; yet, some consumers hold negative perceptions about farmed fish and thus prefer wild-caught selections [40,41]. Rickard et al. [42] identified low levels of knowledge regarding aquaculture products as contributing to negative perceptions, which may in turn limit intake. Further research is needed to determine the extent to which improving the perceptions of available, affordable aquaculture products can contribute to higher levels of seafood intake among U.S. residents in the post-pandemic seafood sector. Since many consumers seem unsure as to whether their purchases included wild-caught or farm-raised selections (or both), an opportunity may exist to promote to consumers the positive attributes of seafood and aquaculture products that would encourage intake. Additional research to confirm which specific attributes consumers value, and whether they are willing to pay a price premium for those attributes, is warranted.

### Study Limitations

The COVID-19 pandemic has been an evolving situation in the U.S., as it is globally. The results presented here represent a cross-sectional assessment of a particular period of time defined as the “early stages” of the COVID-19 pandemic (i.e., March 2020–March 2021). Given the impossibility of projecting a concrete end date to the pandemic and its effects, clear pre- and post-pandemic timelines are difficult to establish for the further analysis and application of our results. It is also recognized that the COVID-19 pandemic has had disproportionate effects on certain segments of the U.S. population that may be influencing dietary behaviors beyond what was captured in this analysis of English-speaking U.S. residents. Future studies should consider conducting surveys in both English and Spanish, and perhaps additional languages, to better capture seafood intake patterns among individuals whose primary language is not English.

An inherent limitation to assessing the impacts of the COVID-19 pandemic on seafood intake is that the circumstances surrounding the pandemic (e.g., policy changes) may have influenced participants’ responses beyond what was measured in this study. Further, systemic inflation observed throughout the food system after the period of data collection for this study may have begun to further impact consumers’ seafood choices. With increasing prices due to inflation [43], consumers’ seafood choices may yet be in a state of flux. A follow-up survey conducted at a time point later in the course of the COVID-19 pandemic could better evaluate the potential impacts of inflation, thus forming a more complete picture of pandemic-related impacts on seafood intake.

Another limitation to the present study is the reliance upon self-reported seafood intake as collected via food frequency recall. Relying on consumer-reported intake, especially over a long time period and during what likely was for many consumers a tumultuous time, may have introduced unmeasurable reporting bias into the study. Future studies might consider incorporating a direct measure of seafood purchases by evaluating household grocery receipts (including in-store and online purchases) or retail point-of-sale data to confirm the types, frequency, and—through the use of bar code data—attributes of the seafood products purchased.

## 5. Conclusions

Many U.S. consumers eat seafood, and most continued to do so during the early stages of the COVID-19 pandemic, whether dining out at restaurants (despite reduced patronage by some) or purchasing seafood for home preparation. Though overall consumer adherence to seafood recommendations increased slightly during the pandemic, most consumers still do not consume enough seafood to maximize its potential health benefits according to the DGA. This phenomenon is widespread across demographic segments, indicating that multiple targeted marketing campaigns and educational interventions may be necessary to effectively increase seafood intake in the U.S. However, the seafood intake deficiency evident among U.S. consumers suggests a strong opportunity for the market growth of various seafood types. Rather than targeting the relatively few non-consumers, broad strategies addressing barriers to intake among current seafood consumers to increase the amount and diversify the types of seafood eaten could increase post-pandemic seafood intake across many consumer segments. Expanding the inclusion and promotion of seafood selections in meal kit and grocery delivery programs may present an effective mechanism to carry this out successfully and represents an area of future study. Further, capitalizing on an interest in seafood as a healthy dietary choice as expressed by some consumers during the COVID-19 pandemic may provide an opportunity to increase the intake of a wider range of seafood types. Given the increased likelihood of DGA adherence among consumers who are familiar with the DGA and those who perceive seafood to be a healthy choice, future research should investigate the effectiveness of specific interventions designed to improve consumer knowledge and health perceptions of seafood. To this end, our research team has an additional study underway to evaluate consumer seafood choices based on product attributes that include health, convenience, and sustainability, all in the context of cost using discrete choice experiments to reflect the reality of the economic influences of seafood production, marketing, sales, and intake.

## Figures and Tables

**Table 1:** Characteristics of U.S. consumers participating in this study (unweighted data).

	U.S. Adults (*N* = 1200)	
	*n*	%
Respondent or Household Member Eats Seafood	1155	96.3%
Gender		
Female	627	52.3%
Male	555	46.3%
Non-Binary/Prefer to Self-Describe *	18	1.5%
Race/Ethnicity		
Non-Hispanic White	816	68.0%
Hispanic	137	11.4%
Non-Hispanic Black	132	11.0%
Asian	42	3.5%
Other	73	6.1%
Education Level Attained		
Less than High School Diploma	41	3.4%
High School Graduate/GED	362	30.2%
College, Less than 4-year Degree	406	33.9%
College, 4-year Degree or Higher	391	32.5%
Marital Status		
Currently Married or Domestic Partnership	668	55.7%
Not Married	532	44.3%
Child(ren) in the Household		
Child(ren) Under 5 years Reside in the Household	87	7.3%
Child(ren) Between 5 and 18 years Reside in the Household	203	16.9%
Prefer Not to Indicate Whether Children Reside in the Household *	12	1.0%
Household Income Level		
$29,999 or less	250	20.8%
$30,000–$59,999	290	24.2%
$60,000–$99,999	270	22.5%
$100,000 and above	271	22.6%
Prefer not to indicate income level	119	9.9%
Coastal or Shoreline State of Residence		
No Coast or Shoreline	234	19.5%
Atlantic Coast	232	19.3%
Pacific Coast	193	16.1%
Gulf Coast	221	18.4%
Great Lakes Shoreline	320	26.7%

* Indicates too few respondents to include in regression analyses.

**Table 2:** Estimated coefficients and marginal effects of predictors for adherence to dietary guidelines among the U.S. general population (*n* = 1004) during the early stages of the COVID-19 pandemic (March 2020–March 2021) based on logistic regression.

	Estimated Coefficient (Standard Deviation)			Marginal Effects (Standard Deviation)			95% Confidence Intervals
Income ($10,000)	0.045 *** (0.008)			0.005 *** (0.001)			0.028–0.061
Male (1/0)	−0.204 (0.201)			−0.024 (0.023)			−0.599–0.190
Married (1/0)	−0.422 *** (0.021)			−0.051 *** (0.004)			−0.463–−0.380
Having child(ren) under 5 years (1/0)	0.273 (0.597)			0.0352 (0.083)			−0.898–1.444
Having child(ren) aged 5–18 years (1/0)	−0.061 (0.265)			−0.007 (0.031)			−0.581–0.459
Race (base = non-Hispanic White)							
Hispanic	0.628 ** (0.263)			0.087 ** (0.044)			0.111–1.144
Non-Hispanic Black	0.919 *** (0.270)			0.139 *** (0.048)			0.390–1.448
Asian	0.895 *** (0.078)			0.141 *** (0.013)			0.743–1.047
Other races	0.773 (0.581)			0.117 (0.105)			−0.366–1.911
Age cohort (base = Silent Generation)							
Baby Boomer	−0.699 *** (0.150)			−0.077 *** (0.017)			−0.992–−0.406
Generation X	−0.299 * (0.164)			−0.034 * (0.018)			−0.620–0.022
Millennial	−0.872 *** (0.115)			−0.091 *** (0.009)			−1.097–−0.648
Generation Z	−0.624 (0.544)			−0.061 (0.045)			−1.691–0.442
Coastal residency (base = no coast/shoreline)							
Atlantic Coast	0.771 *** (0.064)			0.109 *** (0.012)			0.646–0.896
Pacific Coast	−0.119 ** (0.056)			−0.014 ** (0.007)			−0.228–−0.010
Gulf Coast	−0.616 *** (0.221)			−0.064 *** (0.019)			−1.049–−0.183
Great Lakes shoreline	0.467 *** (0.070)			0.060 *** (0.009)			0.329–0.605
Educational attainment (base = High School Graduate or GED)							
Less than High School Diploma	0.146 (0.220)			0.018 (0.028)			−0.285–0.576
College, less than 4-year degree	0.757 *** (0.087)			0.101 *** (0.015)			0.587–0.927
College, 4-year degree or higher	0.811 (0.509)			0.107 (0.076)			−0.186–1.809
Employment (base = not currently employed)							
Currently employed	0.466 ** (0.237)			0.061 * (0.033)			−0.529–1.051
Retired	0.261 (0.403)			0.031 (0.048)			0.002–0.930
Religious affiliation (base = no affiliation, atheist, or agnostic)							
Religion, non-Christian	−0.198 (0.139)			−0.022 (0.014)			−0.471–0.075
Religion, Christian	0.185 (0.186)			0.022 (0.023)			−0.179–0.550
Familiarity with DGA (base = not familiar)							
Some familiarity	0.209 *** (0.045)			0.025 *** (0.006)			0.121–0.298
Very familiar	0.653 ** (0.274)			0.094 ** (0.045)			0.115–1.190
Eats seafood for health	0.164 *** (0.023)			0.020 *** (0.003)			0.120–0.208
Agrees seafood improves heart health	0.485 *** (0.165)			0.054 *** (0.018)			0.161–0.809
Agrees seafood improves blood pressure	0.462 * (0.253)			0.056 * (0.031)			−0.034–0.958
Constant	−3.353 *** (0.174)						
State fixed effects	Yes						
Cluster estimator by coastal vs. non-coastal province							
No. of observations: 1004							
Pseudo-R^2^: 13.76%							
*** *p* < 0.01, ** *p* < 0.05, * *p* < 0.1							
		Actual outcome: meeting DGA (1) or not meeting DGA (0)					
			0		1		
Predicated outcome: meeting DGA (1) or not meeting DGA (0)		0	803		162		
		1	80		26		
Correct prediction ratio			90.94%		13.84%	77.40%	

**Table 3:** Reasons indicated for decreased or increased seafood intake during the early stages of the COVID-19 pandemic (March 2020–March 2021) among U.S. residents who eat seafood (*n* = 1077) (weighted data).

Decreased Seafood Intake	U.S. Residents
	*n* = 151 (13.9%)
Usually eat seafood at restaurants	45.8%
Seafood was more expensive	29.4%
Eating more frequently at home	29.2%
Had less money available	24.7%
Lack of available seafood	15.6%
Difficult to prepare seafood	10.6%
Other	9.0%
Don’t know how to prepare seafood	6.1%
Don’t want seafood in grocery delivery	4.7%
Sustainability reasons	2.6%
Hosting fewer guests	1.6%
**Increased Seafood Intake**	**U.S. Residents**
	***n* = 140 (12.9%)**
Health reasons	44.5%
Seafood is easy to prepare	40.0%
Adding variety to diet	35.1%
Eating more frequently at home	32.7%
Eating more protein	30.7%
Eating more often with seafood eaters	25.6%
Increased availability of seafood	19.5%
Had more money available	18.4%
Found a good seafood recipe	13.0%
Seafood was less expensive	12.3%
Typical meat/poultry was less available	8.1%
Ate at restaurants more frequently	7.4%
Other	6.5%

**Table 4:** Reasons respondents who eat seafood or have a household member who eats seafood (*n* = 1155) did not purchase seafood to prepare at home (weighted data).

Reasons for Not Purchasing Seafood to Prepare at Home	U.S. Residents
	*n* = 189 (16.4%)
I dislike the preparation	26.4%
Other	21.7%
I don’t like the taste	19.9%
The seafood I want is not affordable	17.4%
I don’t like the smell	15.7%
I don’t know how to prepare it	13.1%
My family doesn’t like the taste	9.0%
I don’t know how to select seafood to buy	5.0%
The seafood I want is not available where I shop	4.7%
My family doesn’t like the smell	4.6%

**Table 5:** Reasons respondents who eat seafood or have a household member who eats seafood (*n* = 1155) and ordered groceries and/or meal kits for delivery during the pandemic did not include seafood in their orders (weighted data).

Reasons for Not Including Seafood in Grocery Orders	U.S. Residents
	*n* = 148
I prefer to pick out seafood myself	66.6%
Seafood in my order might be spoiled or damaged	29.5%
Other reasons	13.0%
I personally do not eat seafood	10.5%
**Reasons for Not Purchasing Meal Kits that Contain Seafood**	**U.S. Residents**
	***n* = 25**
I didn’t like the available seafood options	29.1%
Seafood options were not available	24.0%
Seafood in my order might be spoiled or damaged	23.0%
Other reasons	20.6%
I personally do not eat seafood	6.1%

## Data Availability

The data presented in this study are available on request from the corresponding author. The datasets presented in this article are not readily available because aspects of the data are included in ongoing further analyses.
